# Augmented Reality-Based Surgery on the Human Cadaver Using a New Generation of Optical Head-Mounted Displays: Development and Feasibility Study

**DOI:** 10.2196/34781

**Published:** 2022-04-25

**Authors:** Behrus Puladi, Mark Ooms, Martin Bellgardt, Mark Cesov, Myriam Lipprandt, Stefan Raith, Florian Peters, Stephan Christian Möhlhenrich, Andreas Prescher, Frank Hölzle, Torsten Wolfgang Kuhlen, Ali Modabber

**Affiliations:** 1 Department of Oral and Maxillofacial Surgery University Hospital RWTH Aachen Aachen Germany; 2 Institute of Medical Informatics University Hospital RWTH Aachen Aachen Germany; 3 Visual Computing Institute RWTH Aachen University Aachen Germany; 4 Department of Orthodontics Private University of Witten/Herdecke Witten Germany; 5 Institute of Molecular and Cellular Anatomy University Hospital RWTH Aachen Aachen Germany

**Keywords:** digital health in surgery, surgical technique, surgical training, computer-assisted surgery, optical see-through head-mounted display, HoloLens, surgical navigation, medical regulation, open-source, AR, augmented reality, surgery, surgeon, cadaver, serious game, head-mounted display

## Abstract

**Background:**

Although nearly one-third of the world’s disease burden requires surgical care, only a small proportion of digital health applications are directly used in the surgical field. In the coming decades, the application of augmented reality (AR) with a new generation of optical-see-through head-mounted displays (OST-HMDs) like the HoloLens (Microsoft Corp) has the potential to bring digital health into the surgical field. However, for the application to be performed on a living person, proof of performance must first be provided due to regulatory requirements. In this regard, cadaver studies could provide initial evidence.

**Objective:**

The goal of the research was to develop an open-source system for AR-based surgery on human cadavers using freely available technologies.

**Methods:**

We tested our system using an easy-to-understand scenario in which fractured zygomatic arches of the face had to be repositioned with visual and auditory feedback to the investigators using a HoloLens. Results were verified with postoperative imaging and assessed in a blinded fashion by 2 investigators. The developed system and scenario were qualitatively evaluated by consensus interview and individual questionnaires.

**Results:**

The development and implementation of our system was feasible and could be realized in the course of a cadaver study. The AR system was found helpful by the investigators for spatial perception in addition to the combination of visual as well as auditory feedback. The surgical end point could be determined metrically as well as by assessment.

**Conclusions:**

The development and application of an AR-based surgical system using freely available technologies to perform OST-HMD–guided surgical procedures in cadavers is feasible. Cadaver studies are suitable for OST-HMD–guided interventions to measure a surgical end point and provide an initial data foundation for future clinical trials. The availability of free systems for researchers could be helpful for a possible translation process from digital health to AR-based surgery using OST-HMDs in the operating theater via cadaver studies.

## Introduction

Health care is increasingly supported by digital technologies [1]. Almost one-third of the world’s disease burden requires surgical intervention [2], yet only a small fraction of the potential applications of digital health is used in the surgical domain [1]. Current digital health applications such as artificial intelligence (AI)-based predictive models, the use of telemedicine, and wearables do not touch the core of surgical activity in the operating theater [3]. Assistance systems based on augmented reality (AR) or robotics use, on the other hand, would allow the surgeon’s core activities to benefit from digital health in the coming decades [4]. However, the use of autonomous robots in surgery is ambitious considering surgeons take many years to become trained and surgical interventions can often be very situation-specific. In this respect, unlike robotics, AR as an assistance system for the surgeon supports spatial perception and simultaneously incorporates the surgeon’s experience. Thereby, AR-based surgery could be a near future and feasible step toward digital health in the operating theater [5].

Further technical development has made optical-see through head-mounted displays (OST-HMDs) such as the HoloLens (Microsoft Corp) commercially viable with broad use in the industry [6]. Health care and surgery, in particular, are not primarily affected by this development, among other things due to the high regulatory requirements for medical devices. Any researcher in medicine can quickly develop AI-based models with a few lines of script code based on public data and provide proof of performance. However, in surgery with next-generation technologies like AR with OST-HMDs, this development is not yet foreseeable [7].

By feeding back relevant information to the surgeon during surgical tasks based on preoperative or intraoperative medical imaging data or AI-based guided models, AR with or without image-guided surgery (IGS) could overcome one of the main problems of surgical procedures, which is that they mainly rely on the surgeon’s spatial awareness or haptic perception in the surgical field [4,5]. AR itself augments the otherwise real environment with virtual objects, is located in a reality-virtuality continuum, and includes a wide range of technologies [8]. Beside visual perception, AR can also refer to one or multiple combined modalities of perception, such as auditory or haptic [9].

AR applications have been used since the mid-1990s, mainly for surgical procedures on rigid tissue in the head and neck region. Examples of applications in the operating theater are orthognathic surgery, oncology including parotid surgery, and traumatology. Anatomical and pathological structures, drilling and implant position, resection margins, and reconstructive planning are visualized using different AR technologies [10,11]. Similar examples can also be found on cadavers [12-14].

Due to the underlying technology with external monitors, however, many of these deployed systems result in a dissociation between the perceptual site and the operational field [5,10]. HMDs, on the other hand, enable an egocentric view [15] with virtual objects directly displayed in the surgical field of view [10]. Even though HMDs were first described in the 1960s [16], the capabilities of the various HMDs used intraoperatively still vary widely [17]. Basically, 2 classes of AR HMDs can be distinguished [15], optical see-through and video see-through HMDs, the former having the advantage of an unobstructed view of the surgical field [17].

On the road to widespread use of this rapidly developing technology, proof of performance is essential, especially in the regulatory context. Cadaveric studies have long provided a contribution to demonstrating the performance of new medical technologies and are considered a prestudy proof of performance prior to clinical trials [18]. However, cadaveric studies are rare when using OST-HMDs [19-22], and it is still unclear whether cadavers are generally suitable for testing surgical applications with OST-HMDs.

In order to enhance the development of digital health in surgery, we aimed to develop an AR- and OST-HMD–based system for a cadaveric study using free technologies to make it available and adoptable for research in various experimental surgical scenarios as proof of performance. Furthermore, we wanted to investigate if cadaver studies using this system would be suitable for testing system feasibility, applicability in a surgical task that relies primarily on spatial and haptic perception, and evaluability of its surgical end point.

We chose a simple and understandable AR scenario on fresh cadaver heads using a HoloLens, where the surgeon had to reduce a fractured zygomatic arch, a common injury of the human face.

## Methods

### System Development

#### Concept and Requirements

The purpose of our study was to develop an AR-based system for IGS to be used in a surgical environment with human cadavers. The aim was to augment the surgeon’s spatial perception with 3D models based on previous medical imaging by overlaying them on the surgical field using AR. This overlay is intended to be adaptive by adjusting to the current position of the cadaver and surgical instruments and to allow interaction between both. Three essential feedback functions should be provided here: feedback of the proximity of the surgical instrument to surgical target structures by means of a visual signal, an auditory signal, and a visual representation of the movement of the surgical instrument. The graphical user interface should allow intuitive selection of the different cases with specific models and different functions by gestures via AR-based buttons. Furthermore, all described functionalities should also be usable with voice commands to enable hands-free working.

Overall, the system should be easily adaptable to different surgical scenarios, cost-efficient, and easily replicable by third parties, allowing it to be universally applicable as proof of performance for OST-HMDs in surgery on cadavers.

#### Implementation

We aimed to achieve our requirements by using the commercially available HoloLens 1 as one of the state-of-the-art and most broadly used OST-HMDs combined with the camera-based tracking system Vuforia (version 8.5.8, PTC Inc). Our software prototype was developed using the C# programming language with the popular game engine Unity 2018.4.13f LTS (Unity Technologies) and the Mixed Reality Toolkit (version 2.3.0, Microsoft Corp) for rapid prototyping. Our software prototype was then developed into a prerelease candidate of an open-source software as part of a master’s thesis in computer science [23].

**Figure 1:**
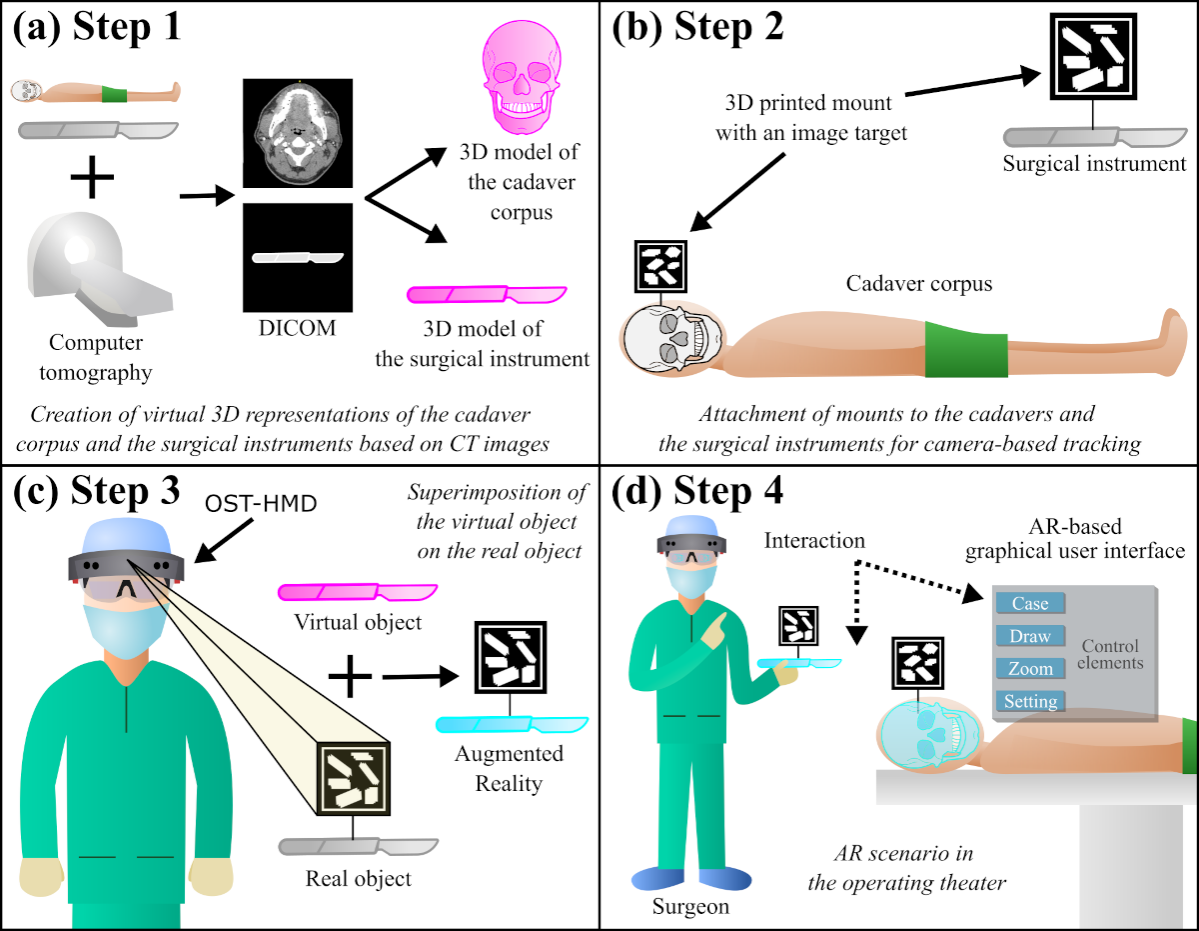
System development: (a) Creation of virtual 3D representations (in purple) of the cadaver and surgical instruments based on computed tomography images. (b) 3D printing of mounts with image targets for attachment to the cadaver and the surgical instruments for camera-based tracking. (c) Superimposition of the virtual 3D models (purple) and real-world object (gray) resulting in an augmented reality (AR) object (cyan). (d) Performing AR-based surgery with an optical see-through head-mounted display. Possibility of interaction between surgical target structures and instruments by means of visual and auditory feedback. Software can be controlled via gestures using an AR-based graphical user interface. DICOM: Digital Imaging and Communications in Medicine; CT: computed tomography; OST-HMD: optical see-through head-mounted display; AR: augmented reality.

**Figure 2:**
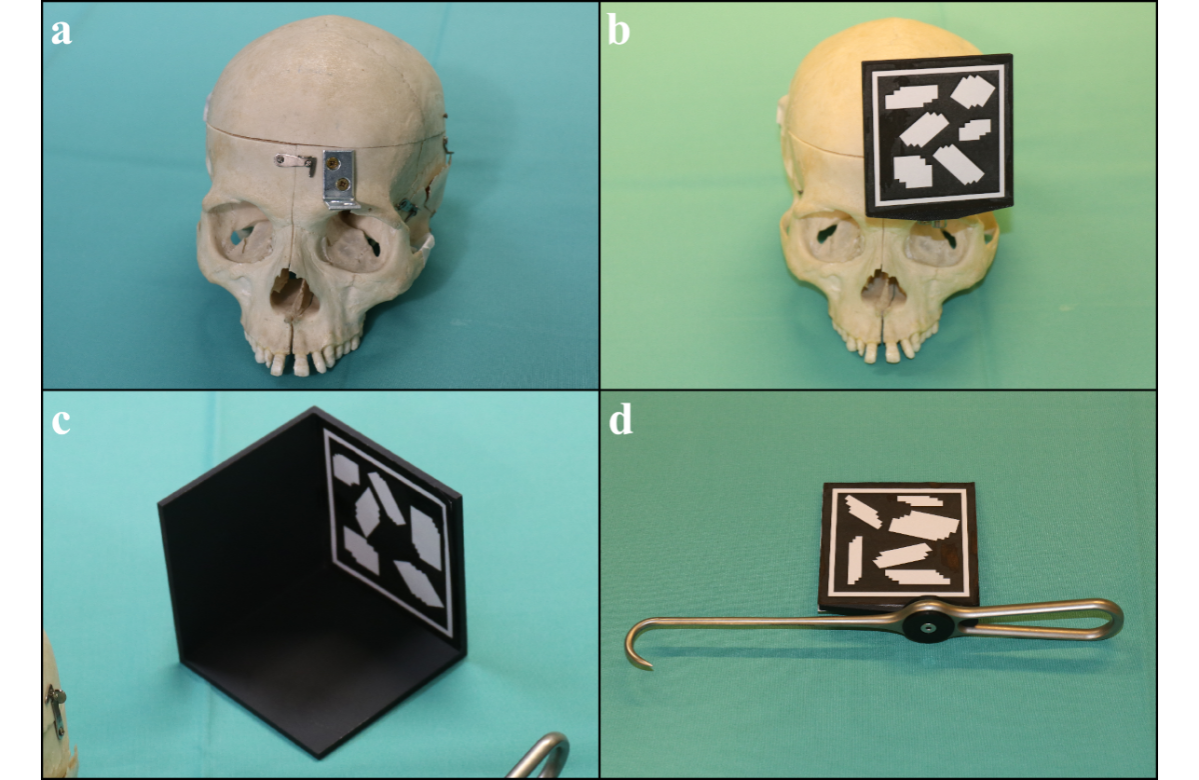
Technical setting: (a) To ensure a consistent method for tracking, a metal angle was attached to the bone of the forehead of each cadaver to attach the tracking mount as not every cadaver head had proper dentition for a stable splint-based tracking. (b) An image target on the mount connected to the forehead via the metal angle. (c) A half-cube for holographic verification can be used for testing the superimposition between real and virtual objects to represent possible errors in the fit of an optical see-through head-mounted display (OST-HMD) or errors in tracking by user-verifiable reference surfaces. (d) The surgical instrument (Stromeyer hook, in our scenario) with an attached tracking mount.

Based on medical imaging data, 3D models were created for the cadavers and surgical instruments (Figure 1a). In order to attach the mounts for image tracking, the cadavers were prepared beforehand to obtain a definite reference point (Figure 1b and Figure 2a). Subsequently, mounts were designed in Autodesk Inventor Professional 2020 (Autodesk Inc), 3D printed using a Fortus 450mc (Stratasys), and attached to the cadaver heads and surgical instruments (Figure 1b and Figure 2b). The image target was used for tracking the cadaver heads (Figure 1c) and for the half-cube for holographic verification described below (Figure 2c). The Vuforia multitarget (corresponds to a combination of image targets so that the surgical instrument can be tracked from both sides) was used for tracking the surgical instrument; in our scenario, a Stromeyer hook (Figure 1c and Figure 2d).

**Figure 3:**
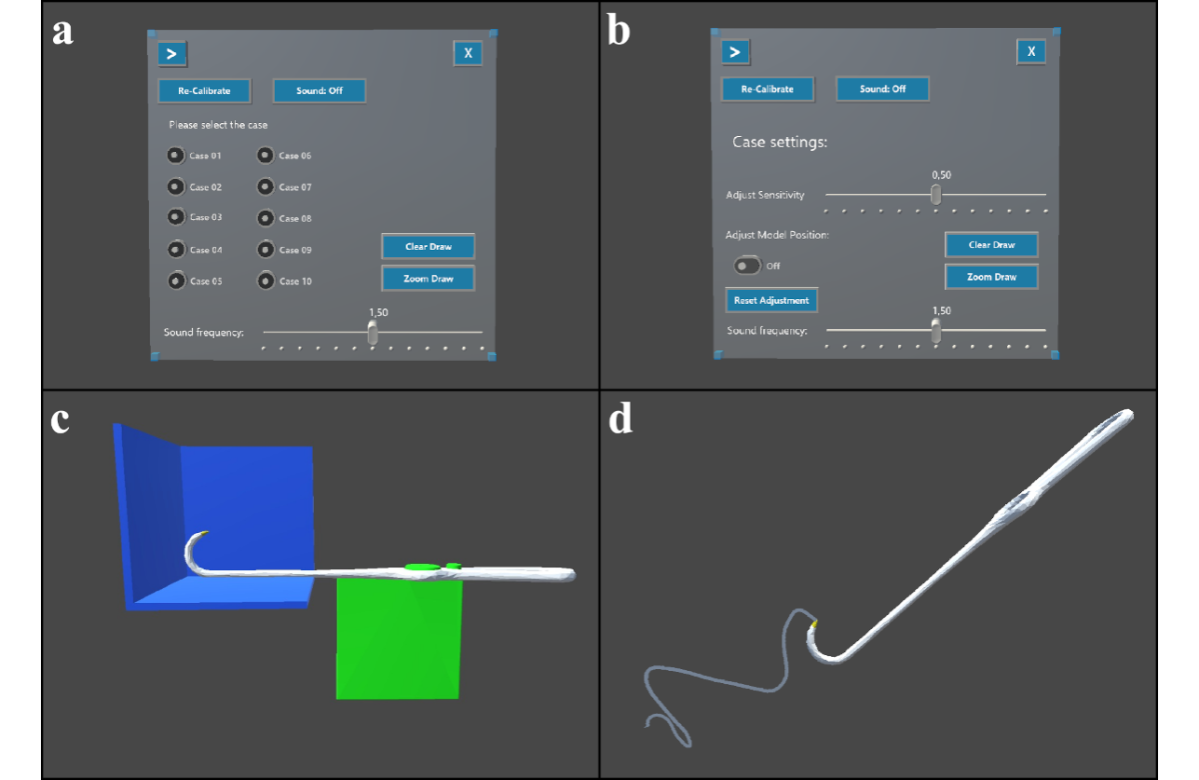
Graphical user interface: (a) Display of the graphical user interface in the Unity development environment. Not all functions mentioned were used in our scenario. The “Recalibrate” button can be used to align the virtual representation of the surgical instrument with the virtual representation of the half-cube. For this, the real surgical instrument (Stromeyer hook) must be exactly aligned with the surfaces of the real half-cube for the holographic verification. Acoustic feedback can be deactivated via the “Sound: Off” button. Additionally, the slider provides an adjustment of the sound functionality depending on the distance [d] and the factor [a] with the formula [d^a^]. To visualize the movement of the surgical instrument, the “Draw” function can be used to display the trajectory by a 3D line. With the button “Enlarge drawing,” the drawing can be zoomed in and with the button “Delete drawing,” the drawing function can be reset. A selection of radio buttons to choose the appropriate cadaver case. (b) Additionally, the virtual and real cadaver head could be adjusted by hand movement, and the sensitivity of the adjustment could be controlled by a slider. This function was not used. (c) Representation of the calibration function with the half-cube in blue, the surgical instrument in white, and the holder for the image target in green. (d) Illustration of the visualization of the instrument trajectory.

**Figure 4:**
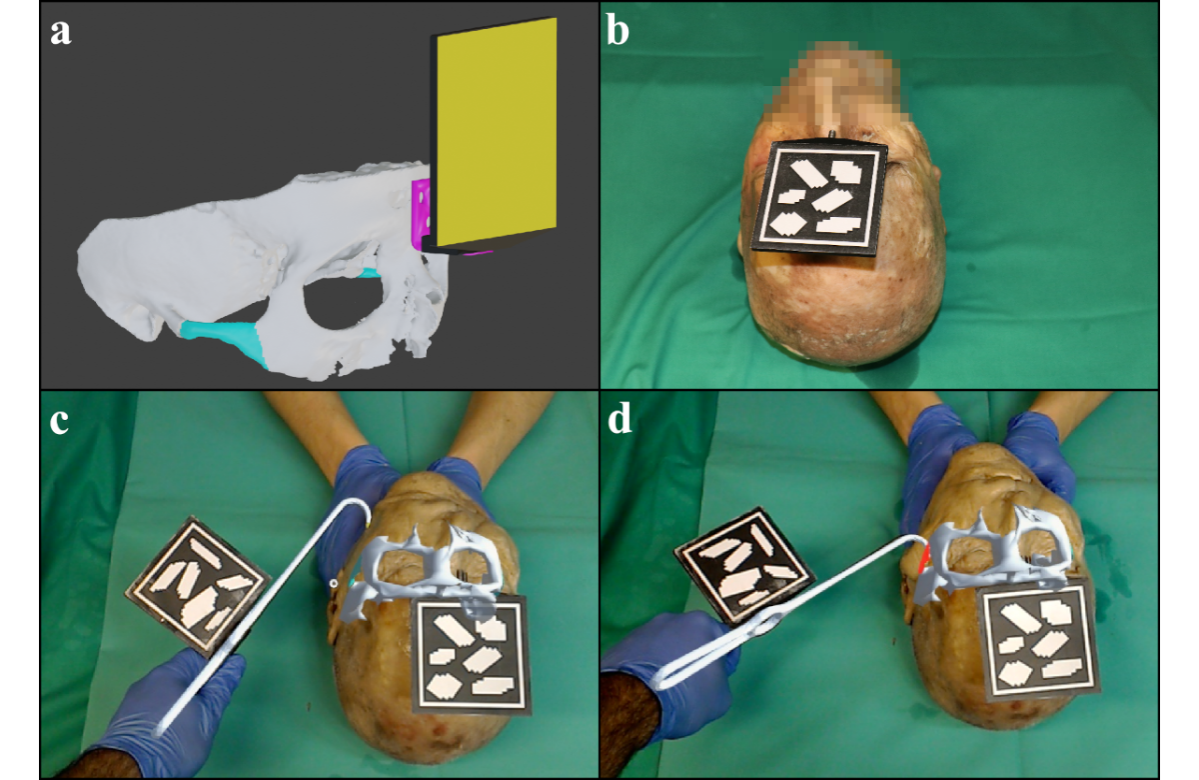
Cadaver trial: (a) A 3D model of the facial skull (white) of one of the cadaver cases with a color representation of the unfractured zygomatic arch (cyan), fixed metal angle (purple), mounting (black), and image target (yellow). (b) A fresh cadaver head shows the placed mount and image target for navigation. (c-d) Photograph taken through HoloLens as one of the investigators performs the cadaver trial. (c) The cadaver head is overlaid with the virtual bone model. The zygomatic arch is shown in green because the tip of the virtual Stromeyer hook has not yet collided with the intended position of the nonfractured zygomatic arch model. The Stromeyer hook is superimposed with an accurate virtual model of itself. (d) The tip of the virtual Stromeyer hook now touches the model of the nonfractured zygomatic arch, resulting in a color change of the zygomatic arch model to red.

A graphical user interface was developed for the AR software to make the virtual anatomical models of the respective cadavers selectable via AR-based buttons and to adjust the software (Figure 1d and Figure 3). It had 3 main functionalities: visual feedback in the region of interest by a color transition of the models from green to red when the tip of the virtual surgical instrument touches the virtual cadaver model (Figure 4c and 4d), auditory feedback from an acoustic tone whose pitch was modulated depending on the distance between the tip of the virtual instrument and the virtual model (Figure 3a and 3b), and visual feedback through virtual drawing (Figure 3d). In our scenario, it was possible to trace the inner contour of the zygomatic arch with the tip of the Stromeyer hook and then visualize it within a bounding box at different sizes and from different directions to evaluate the shape of the inner zygomatic arch contour. All functionalities described were also usable with voice.

When a virtual model was selected, it was superimposed on the real cadaver head by continuous tracking (Figure 1d, Figure 4c, and Figure 4d). The surgical instrument was tracked throughout. To evaluate the perceived superimposition between virtual and real surgical instruments, a half-cube was printed with distinct reference surfaces.

### Ethics Approval

This article does not include studies with live human participants or animals. Approval by the ethics committee of the University Hospital RWTH Aachen (approval number EK 348/21) has been granted. The investigators agreed to participate in the study.

### Cadaveric Trial

#### Preparation

Ten fresh cadaver heads were randomly selected. The initial condition of the facial skeleton was first scanned with cone beam computed tomography (CBCT, Dentsply Sirona). To ensure a consistent method, a metal angle was attached to the bone of the forehead of each cadaver to attach the tracking mount (Figure 2a and 2b) as not every cadaver head had proper dentition for stable splint-based tracking. Subsequently, all zygomatic arches were randomly fractured by a direct blow with a surgical hammer. The fractured state was then scanned again with CBCT. Thereafter, all cadaver heads were frozen until study examination. The resulting fractured zygomatic arches had 1 to 5 fragments. A total of 16 zygomatic arch fractures were classified as type II, 3 as type III, and 1 as type IV, according to Yamomoto et al [24] (Multimedia Appendix 1).

Based on the acquired medical imaging data, 3D models were created for all cadaver heads with the initial situation and fractured zygomatic arches. Both models were registered using the best fit alignment feature of Geomagic Studio 2013 (3D Systems Inc) and loaded into our software. In addition, a Stromeyer hook, a surgical instrument routinely used to reduce zygomatic arch fractures, was digitized and loaded into our software. For tracking, mounts were then 3D printed for fixation on the cadaver heads and on the Stromeyer hook (Figure 2d).

#### Trial

The cadaver heads were randomly assigned to the investigators (a resident and a senior surgeon). One zygomatic arch side of each head was randomly selected (based on a random number generator) for reduction by the conventional method and the opposite side by the AR-based method. Before reduction, the investigators were able to view the CBCT imaging data with the fractured situation on a computer. The conventional reduction was performed with the Stromeyer hook through a percutaneous incision and was based only on haptic perception. The AR-based reduction was performed identically, with the addition of a registered virtual model of the Stromeyer hook and a registered virtual model of the intact zygomatic arch of the corresponding cadaver displayed on the HoloLens. The aforementioned functionalities of feedback through color transition, virtual drawing, and audio signals provided the investigators with additional visual and auditory perception (Figure 4c and 4d). For both methods, the time between the percutaneous incision and performed reduction was measured. After complete reduction, the corresponding cadaver head was scanned with CBCT.

#### Evaluation

**Figure 5:**
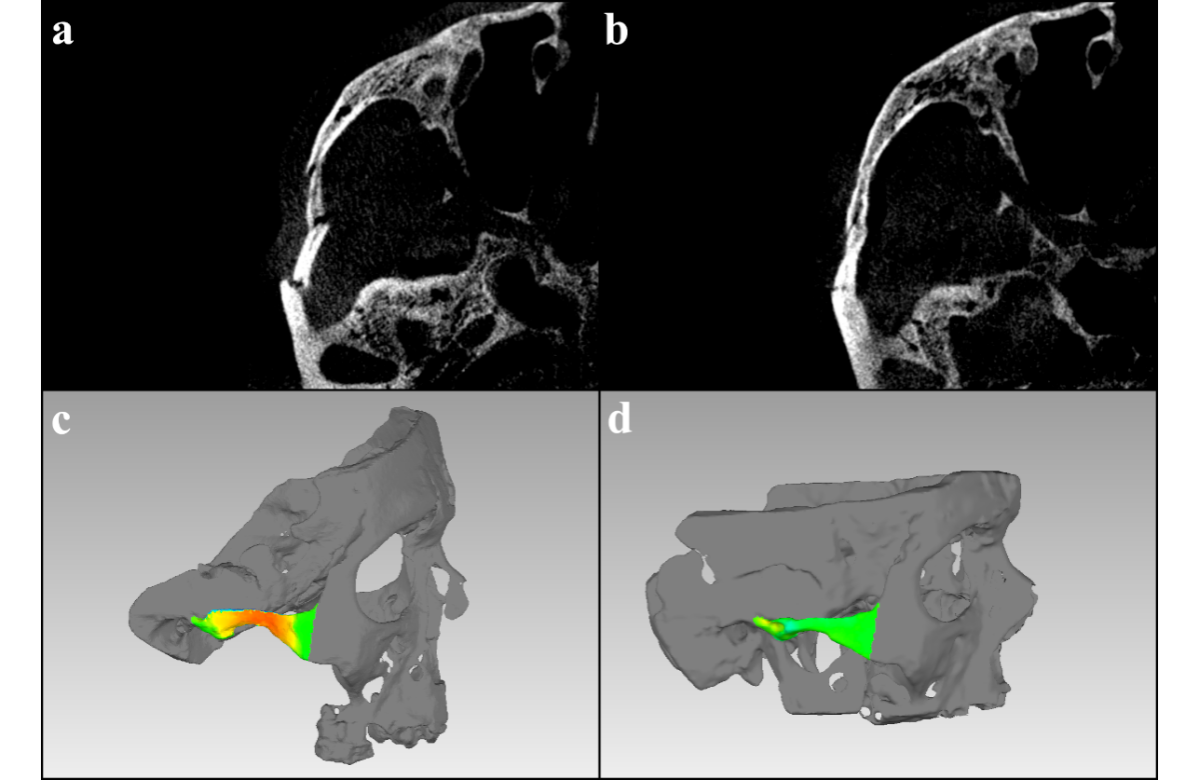
Evaluation: (a) A fractured zygomatic arch visualized before reduction and (b) after reduction in axial cone beam computed tomography slices. (c) The deviation of a fractured zygomatic arch is displayed in color in Geomagic Studio 2013 (3D Systems Inc). Red is for severe deviation (≥1 mm) and green for minor deviation (<1 mm). (d) The same case in Geomagic Studio 2013 after reduction.

Based on postoperative imaging, 3D models of the cadaver heads were created and registered with the corresponding preoperative and initial situation using Geomagic Studio 2013. The zygomatic arch was defined as the region from the temporal origin of the zygomatic process to a straight vertical extension line at the posterior margin of the frontosphenoid process of the zygoma and converted to separate models. The deviation of the different models was then compared (settings: maximum deviation 10 mm, critical angle 45.0°; display resolution set to fine). The initial nonfractured model was used as a reference and compared to the fractured model and subsequently to the reduced model (Figure 5). The results obtained were exported for statistical analysis.

Finally, reduction quality was classified into 4 levels based on postoperative imaging by 2 noninvestigators in a consensus and blinded fashion according to Yakomoto et al [24]: poor for reduction without improvement in bone fragment shape and continuity, fair for incomplete restoration but an improvement in bone fragment shape and continuity, good for near-complete restoration of shape with and without continuity of bone fragments, and excellent for complete restoration of shape with continuity of bone fragments.

The AR software was assessed using the System Usability Scale (SUS) [25]. Afterward, a consensus interview with open-ended questions was conducted with both investigators, and the AR-based scenario was qualitatively assessed using an individual questionnaire (Multimedia Appendix 2) on a 5-point Likert scale (1=strongly disagree; 5=strongly agree).

### Statistical Analysis

The R programming language (R Foundation for Statistical Computing) was used for statistical analysis. Results were expressed as mean and standard deviation. The 95% confidence intervals were calculated by bootstrapping with 1000 replications [26].

## Results

### Surgical Outcome

**Figure 6:**
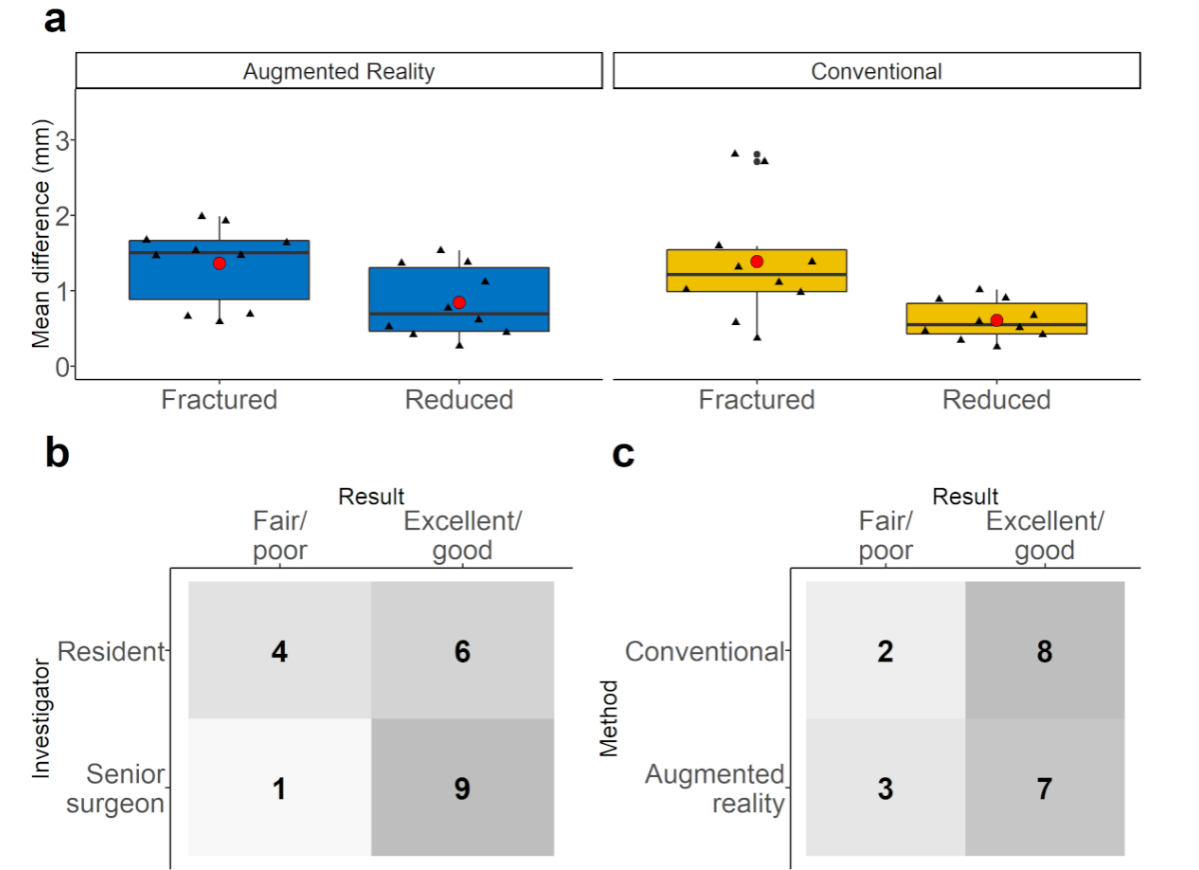
Results: (a) The absolute surface deviation of the fractured and reduced model was calculated in comparison to the nonfractured model and presented as a boxplot before and after reduction for the augmented reality–based method (in blue) and the conventional method (in yellow). Black triangles represent individual measured values. The large red dot represents the mean value and black dots represent outliers. (b,c) Results of zygomatic arch repositioning were determined by 2 investigators in a blinded fashion (for the method) and by consensus. Displayed as a 4-panel chart. Excellent/good was rated as an adequate and fair/poor as an inadequate surgical outcome. (b) Comparison of the resident with the senior surgeon. (c) Comparison based on the method used.

Within the quantitative reduction measurement between fractured and reduced zygomatic arches, our test scenario showed a mean reduction of 0.78 mm (95% CI 0.37-1.29 mm) for the conventional method and 0.52 mm (95% CI 0.23-0.77 mm) for the AR-based method (Figure 6a). The mean time to perform zygomatic arch reduction using the conventional method was 84 seconds (95% CI 52-116 s) and for the AR-based method was 115 seconds (95% CI 54-198 s). A distinct difference in zygomatic arch reduction was observed between the resident and the senior surgeon. Of the 10 zygomatic arch reductions performed by the senior surgeon, 9 were rated good or excellent, while 6 of 10 performed by the resident were rated good or excellent (Figure 6b). This distinct difference was not present between the conventional and AR method, with 8 of 10 zygomatic arch repositions rated good or better for the conventional method and 7 of 10 for the AR method (Figure 6c).

### Evaluation by Investigators

Based on consensus interviews with both investigators, the vertical field of view (FOV) was considered small and tracking mounts could interfere in certain surgical scenarios. When using the HoloLens 1, it was noted that an incorrect fit on the head could also lead to an error in superimposition between virtual and real objects. In this context, the simple half-cube for holographic verification was perceived as helpful for evaluation. Visualization of the fractured condition was preferred over the nonfractured one for navigation.

In addition, an individual Likert questionnaire was performed (Table 1). Both investigators agreed that the holographic visualization of the skeleton by means of an OST-HMD was helpful for spatial perception (mean 4.5) and that it appeared as an integrated part of the fresh cadaver head (mean 4.0). They disagreed that the attached mount for tracking the surgical instrument was perceived as disturbing in that scenario (mean 2.5). Both disagreed with the statement that they felt insecure using the AR-based method (mean 1.5) and agreed that they felt confident using the AR-based method when reducing the zygomatic arch (mean 4.5). They also expressed a preference to use the AR-based method on real patients (mean 4.5) and strongly agreed that they found the AR-based method helpful in the field of haptic surgery (mean 5.0). The average SUS for the AR application was 90 and can thus be rated as best imaginable.

**Table 1:** Questionnaire results^a^.

Item	Resident	Senior surgeon	Mean
1. I found the holographic visualization of the zygomatic arch by means of OST-HMD^b^ helpful for my spatial perception.	4	5	4.5
2. I felt the holographic representation of the zygomatic arch was an integrated part of the cadaver head.	4	4	4.0
3. I found the visual feedback from the color change during the zygomatic arch reduction helpful.	5	4	4.5
4. I found the auditory feedback by changing the tone amplitude during the zygomatic arch reduction helpful.	4	5	4.5
5. I found the drawing function helpful for the visual representation of bone contours.	4	4	4.5
6. I found the navigation holder for the surgical instrument disturbing.	2	3	2.5
7. I think the AR^c^-based method is helpful in haptic surgery.	5	5	5.0
8. I felt more confident in the zygomatic arch reduction using the AR-based method.	5	4	4.5
9. I have felt insecure about the zygomatic arch reduction due to the AR-based method.	1	2	1.5
10. I would like to use the AR-based method on real patients.	5	4	4.5

^a^1=strongly disagree; 5=strongly agree.

^b^OST-HMD: optical see-through head-mounted display.

^c^AR: augmented reality.

## Discussion

### Principal Findings

In our study, we demonstrated that it is possible to develop an adaptable and usable AR-based system with OST-HMDs and image-guided capacities for surgical interventions by combining freely available technologies and evaluating them in a test scenario on human cadavers. This system can be adopted by researchers worldwide and adapted to their own surgical scenarios. The implementation will require a HoloLens or Unity-compatible OST-HMD, the ability to capture 3D medical imaging data, and a 3D printer to produce suitable mounts for the cadaver and surgical equipment. The software and models of the 3D mounts are freely available under an open-source license.

However, the presented system still has shortcomings. When using HoloLens, we have found that an incorrect fit on the head leads to a positioning error between the eyes and the semitransparent display, causing perceptual errors. Only with the correct fit were both real and virtual objects correctly superimposed. For this reason, the simple half-cube we have developed for holographic verification can be used for evaluating the superimposition between real and virtual objects to represent possible errors in the fit of an OST-HMD or, additionally, errors in tracking by user-verifiable reference surfaces (Figure 2c and Figure 3c). The FOV of the HoloLens 1 with 34° was perceived as relatively low [27], although it was judged to be sufficient for our procedure. Depending on the surgical procedure, it could also lead to poor ergonomics and potentially affect the success of the surgical intervention. An enlarged FOV like on the HoloLens 2 [27] could possibly alleviate this.

Although the tracking mount was not found to be a disturbance by the investigators, it could become a potential concern during surgical procedures where space is limited or could lead to tracking errors because the tracking mount is obscured. One solution would be a mount-free or electronic tracking method to avoid disturbing surgeons in such situations [28].

Previous studies that evaluated image-based tracking using Vuforia and a HoloLens indicated a position error of 1.74 to 1.94 mm [29,30]. Our visual evaluation using a printed half-cube conformed to the range of the aforementioned studies (~2 mm). We did not perform a reexamination because we used the same system as the studies mentioned [29,30]. Overall, we considered this sufficient for a majority of surgical scenarios for the first proof of performance. However, the tracking was slow with quick instrument or head movements. This could be improved by increased hardware performance provided by the HoloLens 2 or by holographic remoting, where the main computational load is carried out on an external computer [31]. During the development of our system, we noticed that tracking with Vuforia is faster when the image targets have a black background, which further addressed performance limits [23]. Another alternative would be markerless registration, which has shown an average positioning error between 3.3 to 9.3 mm, depending on the spatial direction. In the future, this error might be reduced with more powerful hardware and could be a serious alternative, especially since markerless registration has no potentially disturbing markers in the surgical field [32]. Manual registration, which showed a mean error of alignment of 12.4 mm, would be another option. After appropriate training or assistance by fiducial markers, the error was reduced to 10 mm [11,33]. Consequently, image-based registration with Vuforia, which is much more accurate, is still the method of choice for most applications [29,30].

In our scenario, it was possible to visualize both the fractured and nonfractured situation, as the healthy bone condition can often be reconstructed with little effort by mirroring the nonfractured side, especially for the face [34]. Since individual bone fragments could not be tracked with our method, we presented the nonfractured situation as a guide for bone reposition. During the consensus interview, this was perceived as a disadvantage for conducting the reduction since the ideal situation can be easily imagined by the investigators themselves. Consequently, the fractured situation and, if necessary, an additional nonfractured situation should be offered for visualization in future examinations. The visualization of the bone with shaded 3D models was perceived as an integral part of the cadaver head. However, further research should focus on whether the use of different display methods such as points, lines, contours, planes, surfaces, wireframes, meshes, and volumes offer advantages in surgical procedures with AR [5].

Our system provided visual and auditory feedback depending on the distance of the surgical instrument working area and intended reduction situation of the bone. This was realized visually via a color change of the virtual zygomatic arch model as well as via the possibility of graphical representation of the movement path of the surgical instrument tip. The drawing function can be used to show the internal contour of bones—in our case, the contour of the fractured bone. If a fracture offset is present, it would be represented by an offset of the drawing line (Figure 3d). To our knowledge, this is the first time that an intraoperative drawing function has been applied in surgery with an OST-HMD. The acoustic feedback operated by increasing the amplitude as a function of the distance between the working area of the surgical instrument and surgical target structure. The combination of visual and auditory feedback was found to be helpful by the investigators. This is consistent with the observation in an AR-based model scenario for a needle biopsy performed by surgeons, where the combination of visual and auditory feedback significantly reduced localization error and increased the success rate [35].

It has already been shown that studies with OST-HMDs on cadavers are suitable to measure the difference between the planning of drill holes, placement of screws, or performance of osteotomies and the actual performance [19-22]. Our study was also able to demonstrate that cadaveric studies with OST-HMDs are suitable to determine fracture reductions quantitatively and qualitatively and thus in one of the veritable surgical end points. In this regard, expected differences between a resident and a senior surgeon were observed. The advantage of using fresh cadavers is the presence of realistic and complex anatomical conditions and thus a situation analogous to the living patient without taking possible surgical risks.

Studies on fresh cadavers, however, cannot determine clinical outcomes such as pain, patient-guided range of motion, dysfunction, or other clinical parameters. Nevertheless, cadaveric studies can be used to provide a data basis for subsequent clinical study planning. The technical system can be evaluated and tested. The developed AR system did not result in a large temporal difference from the conventional method in our scenario. It is important to measure duration as an end point, as surgery time is an important quality indicator. Prolonged surgery durations lead to a greater number of complications for the patient [36] and increased costs for the health care system [37]. Furthermore, the quantitative (reduction in mm) and qualitative (assessed reduction quality) data obtained can be used to plan the sample size for larger cadaveric studies or clinical trials. Procedures where the number of subjects to be treated according to sample size planning is already very large and thus the effect is at the same time very weak may therefore not add much value and could be avoided in this way.

Overall, the number of studies with application of AR-based surgery with OST-HMDs in cadaveric studies is small [19-22]. In contrast, AI-based models can be developed by any researcher today with public data and a few lines of scripting. By this method, breakthrough results in diagnostics and nonsurgical therapy were achieved. However, a similar development for surgery that digitizes the operation field is missing. For this to happen, AR-based applications must become mass-market ready and proof of performance must be provided. We hope that other researchers will feel motivated to develop their cadaver test scenarios with this prototype system.

### Conclusion

The development and application of an AR-based surgical system using freely available technologies to perform OST-HMD–guided surgical procedures in cadavers is feasible, but our presented open-source prototype should be further developed. Cadaver studies are suitable for OST-HMD–guided interventions to measure a surgical end point and provide an initial data foundation for future clinical trials. In this regard, it has been shown in our scenario that the effect of the AR-based approach could be more likely to make a difference in residents. This should be considered when planning future trials. The availability of free systems for researchers could be helpful for a possible translation process from digital health to AR-based surgery using OST-HMDs in the operating theater via cadaver studies.

## Appendix

Multimedia Appendix 1 Overview of zygomatic arch fractures.

Multimedia Appendix 2 Questionnaire.

## Abbreviations

AI: artificial intelligence
AR: augmented reality
CBCT: cone beam computed tomography
FOV: field of view
IGS: image-guided surgery
OST-HMD: optical see-through head-mounted display
SUS: System Usability Scale
