# Supplementary material for: Augmented Reality-Based Surgery on the Human Cadaver Using a New Generation of Optical Head-Mounted Displays: Development and Feasibility Study
Source: JMIR Serious Games. 2022 Apr 25;10(2):e34781. doi: 10.2196/34781 (PMC9086879; doi:10.2196/34781)
Supplement: Multimedia Appendix 1 [file games_v10i2e34781_app1.docx]

## Multimedia Appendix 1. Overview of zygomatic arch fractures.

| **# Zygomatic Arch** | **Side** | **Fracture** | | **Classification** | **Method** | **Investigator** | **Reduction status** |
| --- | --- | --- | --- | --- | --- | --- | --- |
|  |  | **Fragments** | **Lines** |  |  |  |  |
| 1 | Left | 2 | 3 | II | AR | Senior surgeon | Good |
| 2 | Left | 1 | 2 | II | AR | Senior surgeon | Excellent |
| 3 | Left | 1 | 2 | II | AR | Senior surgeon | Good |
| 4 | Right | 2 | 3 | II | AR | Senior surgeon | Fair |
| 5 | Right | 4 | 5 | II | AR | Senior surgeon | Excellent |
| 6 | Left | 2 | 3 | III | AR | Resident | Fair |
| 7 | Right | 2 | 3 | IV | AR | Resident | Good |
| 8 | Left | 1 | 2 | II | AR | Resident | Excellent |
| 9 | Left | 2 | 3 | II | AR | Resident | Good |
| 10 | Right | 2 | 3 | III | AR | Resident | Fair |
| 11 | Right | 1 | 2 | III | Conventional | Senior surgeon | Good |
| 12 | Right | 2 | 3 | II | Conventional | Senior surgeon | Excellent |
| 13 | Right | 2 | 3 | II | Conventional | Senior surgeon | Good |
| 14 | Left | 2 | 3 | II | Conventional | Senior surgeon | Excellent |
| 15 | Left | 2 | 3 | II | Conventional | Senior surgeon | Good |
| 16 | Right | 2 | 3 | II | Conventional | Resident | Excellent |
| 17 | Left | 5 | 6 | II | Conventional | Resident | Good |
| 18 | Right | 1 | 2 | II | Conventional | Resident | Fair |
| 19 | Right | 2 | 3 | II | Conventional | Resident | Excellent |
| 20 | Left | 1 | 2 | II | Conventional | Resident | Fair |

**Supplementary Table 1** **Overview of zygomatic arch fractures.** (#) Numbering of zygomatic arch fractures. (Side) side of the zygomatic arch. (Fracture) number of bone fragments and fracture lines; (Classification) Classification according to Yamomoto et al. ^20^: Type II: Displacement without bone contact at 1 fracture line; Type III: Displacement without bone contact at 2 fracture lines; Type IV: Displacement without bone contact at 2 fracture lines. (Method) Augmented reality-based or conventional-based method. (Reduction status): Reduction status according to Yakomoto et al. ^20^: Poor for reduction without improvement in bone fragment shape and continuity, fair for incomplete restoration but an improvement in bone fragment shape and continuity, good for near-complete restoration of shape with and without continuity of bone fragments and excellent for complete restoration of shape with continuity of bone fragments.
