# Supplementary material for: Augmented Reality-Based Surgery on the Human Cadaver Using a New Generation of Optical Head-Mounted Displays: Development and Feasibility Study
Source: JMIR Serious Games. 2022 Apr 25;10(2):e34781. doi: 10.2196/34781 (PMC9086879; doi:10.2196/34781)
Supplement: Multimedia Appendix 2 [file games_v10i2e34781_app2.docx]

## Multimedia Appendix 2. Questionnaire.

|  | **Strongly**  **disagree** | |  | | | **Strongly**  **agree** | |
| --- | --- | --- | --- | --- | --- | --- | --- |
|  | **\|---------------\|---------------\|---------------\|---------------\|** | | | | | | |
| I found the holographic visualization of the zygomatic arch by means of OST-HMD helpful for my spatial perception. | ⬜ | ⬜ | | ⬜ | ⬜ | | ⬜ |
| I felt the holographic representation of the zygomatic arch was an integrated part of the cadaver head. | ⬜ | ⬜ | | ⬜ | ⬜ | | ⬜ |
| I found the visual feedback from the color change during the zygomatic arch reduction helpful. | ⬜ | ⬜ | | ⬜ | ⬜ | | ⬜ |
| I found the auditory feedback by changing the tone amplitude during the zygomatic arch reduction helpful. | ⬜ | ⬜ | | ⬜ | ⬜ | | ⬜ |
| I found the drawing function helpful for the visual representation of bone contours. | ⬜ | ⬜ | | ⬜ | ⬜ | | ⬜ |
| I found the navigation holder for the surgical instrument disturbing. | ⬜ | ⬜ | | ⬜ | ⬜ | | ⬜ |
| I think the AR-based method is helpful in haptic surgery. | ⬜ | ⬜ | | ⬜ | ⬜ | | ⬜ |
| I felt more confident in the zygomatic arch reduction using the AR-based method. | ⬜ | ⬜ | | ⬜ | ⬜ | | ⬜ |
| I have felt insecure about the zygomatic arch reduction due to the AR-based method. | ⬜ | ⬜ | | ⬜ | ⬜ | | ⬜ |
| I would like to use the AR-based method on real patients. | ⬜ | ⬜ | | ⬜ | ⬜ | | ⬜ |
